# Netrin-1 controls inflammation in response to ischemic stroke through altering microglia phenotype

**DOI:** 10.3389/fimmu.2023.1178638

**Published:** 2023-06-14

**Authors:** Xiaosheng Yang, Yang Liu, Weijie Zhong, Yi Li, Wenchuan Zhang

**Affiliations:** ^1^ Department of Neurosurgery, Shanghai Ninth People’s Hospital, Shanghai Jiao Tong University, School of Medicine, Shanghai, China; ^2^ Department of Ultrasound, Shanghai General Hospital, Shanghai Jiao Tong University, School of Medicine, Shanghai, China

**Keywords:** ischemic stroke, Netrin-1, microglia, UNC5a, inflammation, neuroprotection

## Abstract

**Introduction:**

The current approaches that are used to treat ischemic stroke suffer from poor targeting, lack of effectiveness, and potential off-target effects, necessitating the development of new therapeutic strategies to enhance neuronal cell survival and regeneration. This study aimed to investigate the role of microglial Netrin-1 in ischemic stroke, a topic that has not been fully understood.

**Methods:**

Netrin-1 levels and its primary receptor expressions were investigated in cerebral microglia from acute ischemic stroke patients and age-matched control subjects. A public database (GEO148350), which supplied RNAseq results for rat cerebral microglia in a middle cerebral artery occlusion (MCAO) model, was analyzed to assess the expression of Netrin-1, its major receptors, and genes related to macrophage function. A microglia-specific gene targeting approach and a delivery system allowing for crossing the blood-brain barrier were applied in a mouse model for ischemic stroke to investigate the role of microglial Netrin-1. Netrin-1 receptor signaling in microglia was observed and the effects on microglial phenotype, apoptosis, and migration were analyzed.

**Results:**

Across human patients, rat and mouse models, activation of Netrin-1 receptor signaling was mainly conducted *via* its receptor UNC5a in microglia, which resulted in a shift in microglial phenotype towards an anti-inflammatory or M2-like state, leading to a reduction in apoptosis and migration of microglia. Netrin-1-induced phenotypic change in microglia exerted protective effects on neuronal cells *in vivo* during ischemic stroke.

**Conclusion:**

Our study highlights the potential of targeting Netrin-1 and its receptors as a promising therapeutic strategy for promoting post-ischemic survival and functional recovery.

## Introduction

Ischemic stroke is a prevalent type of stroke that causes death and disability on a global scale (1). Although recombinant tissue plasminogen activator (rtPA) is the most common drug used for treating ischemic stroke, its effectiveness on thrombolysis and neuroprotection is limited due to a narrow time window and potential risks (2). Despite extensive research, many neuroprotective drugs have failed in clinical trials due to poor targeting, a lack of effectiveness and potential off-target effects (3). Therefore, new therapeutic strategies are required to enhance neuronal cell survival and regeneration following ischemic stroke.

Microglia function as sensor cells in the central nervous system’s immune system (4). Together with infiltrating macrophages, microglia play a crucial role in regulating the brain’s immune and inflammatory response after ischemic injury (5). Studies indicate that microglia and macrophages display distinct functions and phenotypes during ischemic brain injury and can modify their phenotype and function in response to changes in their microenvironment (6, 7). Two known phenotypes are the classical activation phenotype (M1), which is proinflammatory, and the alternative activation phenotype (M2), which is anti-inflammatory. The M2 phenotype promotes tissue repair and regeneration after cerebral ischemia, while the M1 phenotype releases proinflammatory cytokines that aggravate tissue injury. It is very important for microglia to be effectively polarized towards the M2-like phenotype in order to reduce inflammatory damage after a stroke (8).

In the developing nervous system, the guidance of migrating cells and axons to their targets is mediated with different proteins, including Netrin-1 that works as either a chemoattractant or chemorepellent (9). Several receptors for netrin-1 have been identified (9), including Depleted in Colorectal Cancer (DCC), Unc-5 netrin receptor A (UNC5a), UNC5b and UNC5c (10).

Neuronal Netrin-1 and its receptors have been shown to play a crucial role in post-ischemic survival (11). Acting as an axon guidance molecule, Netrin-1 is able to regulate nervous system development and repair (11). Moreover, Netrin-1 has neuroprotective effects in the ischemic brain, through binding to its receptors to activate various signaling pathways that regulate cell survival and death (12). To this end, Netrin-1 promotes cell survival, reduces neuronal death, and improves functional recovery after ischemic injury (11). While the role of neuronal Netrin-1 in brain development and post-ischemic survival is well-established, the specific function of microglial Netrin-1 in ischemic stroke is not yet clear and thus addressed in the current study.

## Materials and methods

### Ethic issues

The experiments in this study were applied according to the approved protocols by the Institutional Animal Care and Use Committee situated in Shanghai Ninth People’s Hospital. Human specimens of acute ischemic brain tissue and normal brain tissue were collected from deceased patients with the approval of the Ethic department at the Shanghai Ninth People’s Hospital. The study was conducted in compliance with all relevant ethical guidelines and regulations. Patients or their family members provided written approval for the use of their tissue samples in research. The collection of brain tissue was performed postmortem, and the tissue samples were immediately stored at -80°C until use. All measurements and studies were conducted in a blinded and randomized manner.

### Animal work

The C57/Bl6 mice from Charles River Laboratories were housed individually in a climate-controlled environment. Only male mice were used in the experiments. Prior to each experiment, power calculations were conducted to determine the appropriate number of animals required to achieve a statistical significance of P<0.05. Assurance of randomization of mouse grouping was made through an allocation concealment method. To minimize the potential confounding effects of genetic variability, inbred littermate mice were used in each specified experiment. During the analysis, no data were excluded. To generate ischemic stroke, middle cerebral artery occlusion (MCAO) was performed on mice under 1.2% isoflurane anesthesia. The anesthetized mouse was positioned in a stereotaxic frame and a surgical procedure was performed. The procedure includes clamping of the common carotid artery to halt blood flow and insertion of a nylon filament *via* the external carotid artery into the internal carotid artery, ultimately reaching the middle cerebral artery. The filament was left in place for 60 minutes to induce the ischemic insult, and then removed to restore blood flow.

### Behavioral tests

The Neurological Severity Score (mNSS) was used for the assessment of neurological function in mice following MCAO surgery. The mNSS test was composed of several individual tests evaluating different neurological functions, including motor function, sensory function, and reflexes. Briefly, mice were placed on a flat surface and examined for their ability to move their forelimbs and hindlimbs as an indicator for motor function. The mice were given a score from 0-6, with 0 representing normal motor function and 6 representing no movement. Next, the mouse forelimbs and hindlimbs were touched with a soft brush and examined for their response as an indicator for sensory function. The mice were given a score from 0-2, with 0 representing normal sensory function and 2 representing no response. Finally, the mouse forelimbs and hindlimbs were tapped and examined for their response as an indicator for reflexes. The mice were given a score from 0-2, with 0 representing normal reflexes and 2 representing no response. The scores from each of these tests were then added together to give a total mNSS score for each mouse. Higher scores indicate greater neurological deficits, while lower scores indicate better neurological function.

### Infarct size assessment

After inducing ischemic stroke in mice, the brain was dissected three days later and sliced into coronal sections that were 2-mm thick. These sections were then stained with a 2% solution of 2,3,5-triphenyltetrazolium chloride (TTC) and incubated at 37°C for 25 minutes. TTC staining distinguishes infarcted tissue, which appears white due to the loss of mitochondrial dehydrogenase activity, from viable tissue, which appears red. The infarct area was then measured on each slice using ImageJ and the infarct volume was calculated by integrating the areas of all slices.

### Cell lines

To conduct the current research, various cell lines were procured from the American Type Culture Collection (ATCC). These cell lines comprised HMC3, a microglia cell line from mice, RAW264.7, a mouse macrophage cell line, and HCN-2, a mouse neuronal cell line. All the cell lines were cultured in a 5% CO2 atmosphere at 37°C using Dulbecco’s modified Eagle medium (DMEM) supplemented with 8-10% fetal bovine serum (FBS, Sigma-Aldrich).

### Plasmids and adeno-associated virus

A plasmid pUCmini-iCAP-PHP.eB (#103005) was purchased from Addgene, and another microglia-specific TMEM119 promoter (#34837) was purchased from GeneCopoeia. Both plasmids were utilized for production of an adeno-associated virus (AAV) serotype PHP.B vector for the experiment. To allow for controlled expression under the TMEM119 promoter, a green fluorescent protein (GFP) reporter was linked to the Netrin-1 or scramble sequence (Scr) *via* a f2A sequence. Transfection to generate AAVs was performed using Lipofectamine 3000 reagent (Invitrogen) as per the manufacturer’s instructions. For tail vein injection, a single dose of 5X10^12^ viral particles in 100 µL total volume was administered through the mouse tail.

### Quantitative reverse transcription polymerase chain reaction

The expression level of specific genes in sorted microglia from ischemic hemispheres of mice after cerebral ischemia was measured using quantitative reverse transcription polymerase chain reaction (RT-qPCR). Prior to RT-qPCR analysis, total RNAs were extracted and reverse transcribed into cDNA using the high-capacity RNA-to-cDNA kit from ThermoScientific. Qiagen, a reputable supplier of high-quality qPCR reagents, provided the primers for the RT-qPCR analysis. To ensure accurate quantification of gene expression levels, the obtained values were normalized sequentially against β-actin, a widely used reference gene, and experimental controls. Real-time detection of the amount of double-stranded DNA synthesized during PCR was achieved using the SYBR Green method, which takes advantage of its ability to bind to double-stranded DNA and fluoresce when excited by light. This allowed for precise monitoring of the PCR amplification in real-time.

### Flow cytometry

Microglia were sorted from the ipsilateral hemisphere of mice 3 days after cerebral ischemia. The tissue pieces were cut into small fragments and incubated at 37°C for 15 minutes with a pre-warmed enzyme mix (Neural Tissue Dissociation Kit, Miltenyi Biotec). The single cells were labeled with FITC-conjugated anti-TMEM119 and APC-conjugated anti-CD45 antibodies (Becton-Dickinson Biosciences) and analyzed using FACS sorting. The microglia population (CD11b positive and CD45 intermediate positive) was sorted and analyzed using the FlowJo software (Flowjo LLC). For analyzing apoptosis by flow cytometry, cells were resuspended in a buffer containing Annexin V conjugated with GFP and propidium iodide (PI). Annexin V binds to phosphatidylserine (PS) that translocates to the outer leaflet of the plasma membrane during apoptosis, while PI enters cells with compromised cell membranes such as necrotic cells. Afterward, flow cytometry was employed to assess the proportion of cells that were either in the early apoptotic phase (Annexin V positive, PI negative) or in the late apoptotic or necrotic stage (Annexin V positive, PI positive). Controls were also included for unstained cells and those that were separately stained with Annexin V and PI.

### TUNEL staining

The terminal deoxynucleotidyl transferase dUTP Nick-End Labeling (TUNEL) protocol utilizes the *In Situ* Cell Death Detection Kit to detect DNA fragmentation in apoptotic cells (Invitrogen). Fixed and permeabilized tissue sections are incubated with a reaction mixture containing terminal deoxynucleotidyl transferase (TdT) and labeled nucleotides from the kit. TdT incorporates the labeled nucleotides at the fragmented DNA ends. Subsequently, the labeled DNA is visualized using fluorescence or chromogenic detection methods provided in the kit, allowing for the identification of apoptotic cells.

### Enzyme-linked immunosorbent assay, histology, and immunohistochemistry

The mouse brain tissues were fixed with formalin and embedded in paraffin. The counterstaining of hematoxylin was conducted. The standard method of peroxidase-conjugated streptavidin and the substrate 3,3’-diaminobenzidine (DAB) were used sequentially. Immunostaining was carried out with rabbit anti-Netrin-1 antibody (Abcam) or rabbit anti-PHF1 antibody (Abcam) or rabbit anti-TMEM119 (Abcam). PHF-1 positive neuronal cells were quantified for assessing neuronal cell loss. Enzyme-linked immunosorbent assay (ELISA) was performed to measure the levels of mouse Netrin-1 (ELM-NTN1; RayBiotech), mouse DCC (ELM-DCC; RayBiotech), mouse UNC5a (abx552620, Abbexa Ltd, Cambridge, UK), mouse UNC5b (abx552623, Abbexa Ltd), mouse UNC5c (abx520698, Abbexa Ltd), mouse IL-1β (ab197742; Abcam), mouse A2B adenosine receptor (A2B; STJE0006946, St John’s Laboratory, London, UK), mouse IL-6 (M6000B; R&D System), mouse tumor necrosis factor alpha (TNFα, ab208348; Abcam), mouse interferon gamma (IFNɣ, ab282874; Abcam), mouse IL-10 (M1000B; R&D System), and mouse arginase 1 (ARG1, ab269541; Abcam). Specific kits were utilized as per the manufacturer’s instructions.

### Cell viability and scratch cell migration assays

To assess cell proliferation, viable cell counts were measured using the Cell Counting Kit-8 (CCK-8) assay (CCK-8, Sigma-Aldrich). For scratch cell migration assay, a confluent layer of microglia was first grown on a culture dish. A scratch was then created on the cell layer using a pipette tip. The dish was then washed to remove any detached cells and fresh media was added. The cells were then allowed to migrate into the scratch area and the degree of closure or filling of the scratch was measured at 24 hours to quantify the migratory potential of the cells. The migration was measured in the presence or absence of recombinant rat UNC5a-Fc (6767-UN-050; R&D Systems) or whole IgG as control (Jackson Lab).

### Bioinformatics

We examined published databases on rodent ischemic stroke studies and selected the GEO148350 database, which provided RNAseq results for rat cerebral microglia in an MCAO model, for the current study. Data analysis was performed using the GEO2R online bioinformatics tools with R language coding to obtain individual values. Quality control measures included a boxplot representing gene expression data distribution, and a uniform manifold approximation and projection (UMAP) plot to evaluate variable distribution across compared groups. A mean-difference plot was generated to display average gene expression levels between the two groups, with the difference shown on the Y-axis based on a predetermined statistical threshold. Lastly, we generated a volcano plot to identify differentially expressed genes between the groups, displaying the log2 fold change in gene expression levels on the X-axis and the corresponding -log10 p-value on the Y-axis.

### Statistics

All experiments were statistically analyzed using GraphPad Prism 7 software. One-way analysis of variance (ANOVA) was applied to the obtained data, and Bonferroni correction was employed. Where required, Fisher’s exact test was utilized. At least five independent observations were conducted, and data are presented as individual values together with mean ± standard deviation (SD). Statistic significance was considered when P < 0.05.

## Results

### Acute ischemic stroke patients exhibit increased cerebral microglia expressing high Netrin-1 and UNC5a

**Figure 1:**
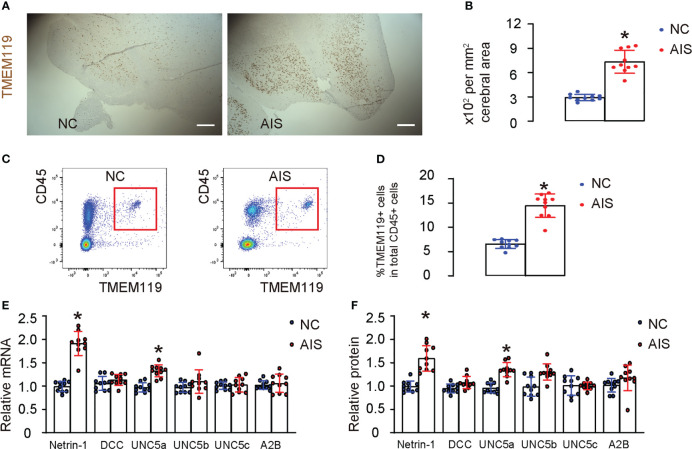
Acute ischemic stroke patients exhibit increased cerebral microglia expressing high Netrin-1 and UNC5a. **(A)** Immunostaining for TMEM119 in brain tissue from patients who had died from acute ischemic stroke (AIS), as well as from age-matched normal cases (NC). **(B)** Quantification of the number of TMEM119-postive microglia in the brain. **(C–F)** Microglia were isolated from AIS and NC brain tissue using flow cytometry, for which microglia were determined and sorted out based on their co-expression for TMEM119 and CD45, shown by representative flow charts **(C)**, and by quantification **(D)**. **(E, F)** RT-qPCR **(E)** and ELISA **(F)** for Netrin-1 and its main receptors, which include DCC, UNC5a, UNC5b, UNC5d and A2B, in isolated microglia. *p<0.05. Scale bars were 100µm.

While the role of neuronal Netrin-1 in brain development and post-ischemic survival is well-established, the specific function of microglial Netrin-1 in ischemic stroke is not yet clear. In order to investigate this question, we used immunostaining to detect microglia-specific marker TMEM119 in brain tissue from patients who had died from acute ischemic stroke (AIS), as well as from age-matched normal cases (NC). We found a significantly greater number of TMEM119-positive microglia in the AIS tissue compared to the NC tissue (**Figures 1A, B**). Next, microglia were isolated from AIS and NC brain tissue using a flow cytometry-based strategy, for which microglia were determined and sorted out based on their co-expression for TMEM119 and CD45, a marker for microglia and a marker for leukocytes, respectively (**Figure 1C**). Neurons and other non-inflammatory cells lack expression of CD45. On the other hand, granulocytes, macrophages, lymphocytes, and dendritic cells express high levels of CD45 but not TMEM119. Our results showed significantly more microglia in the AIS compared to NC (**Figure 1D**), consistent with our immunostaining data (**Figures 1A, B**). Next, we examined the expression of Netrin-1 and its main receptors, which include DCC, UNC5a, UNC5b, UNC5d and A2B adenosine receptor (A2B), in isolated microglia. Our results revealed a significant increase in the expression of Netrin-1 and UNC5a, but not DCC, UNC5b, UNC5d, and A2B in microglia from the brains of AIS compared to those from the brains of NC (**Figures 1E, F**). These findings indicate that cerebral microglia from patients with ischemic stroke exhibit elevated expression of Netrin-1 and its receptor UNC5a.

### Rodent study demonstrates upregulation of Netrin-1 and its receptor UNC5a in microglia after ischemic stroke

**Figure 2:**
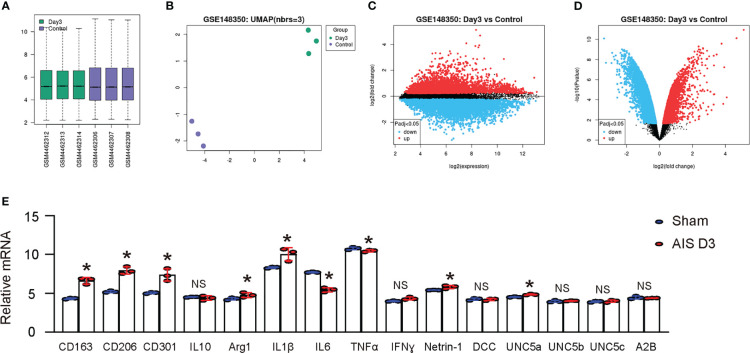
Rodent study demonstrates upregulation of Netrin-1 and its receptor UNC5a in microglia after ischemic stroke. A published database (GEO148350) provided RNAseq results on rat cerebral microglia in an MCAO model. We analyzed these data using GEO2R online bioinformatics tools with R language coding to extract individual values. We focused on microglia isolated from young rats (3 months of age) at day 3 after induction of ischemia and from sham treatment, which were most relevant to our study. **(A)** A boxplot. **(B)** A UMAP plot. **(C)** A mean-difference plot. **(D)** A volcano plot. **(E)** Array reads for genes related to microglia polarization and Netrin-1 receptor signaling. *p<0.05. NS, no significance.

Then, we searched published databases on rodent studies of ischemic stroke and found a database (GEO148350) that provided RNAseq results on rat cerebral microglia in an MCAO model. We analyzed these data using GEO2R online bioinformatics tools with R language coding to extract individual values. We utilized data from microglia isolated from young rats (3 months old) at day 3 post-ischemia induction and sham treatment, as it passed quality control measures including not only a boxplot displaying gene expression data distribution for each group (**Figure 2A**), but also a UMAP plot to assess variable distribution across the compared groups (**Figure 2B**). Then, we generated a mean-difference plot to display the average gene expression levels for the sham and day 3 groups, with the difference between the two groups shown on the Y-axis (**Figure 2C**). Finally, we generated a volcano plot to identify genes that were significantly up- or down-regulated in the day 3 group compared to the sham group (**Figure 2D**). Next, we first looked at genes associated with macrophage polarization. Notably, in rat microglia following D3 AIS induction, the M2-related markers CD163, CD206, CD301, and arginase 1 exhibited significant increases, while the M2-related factor IL10 did not show a significant change. Among the M1 markers, IL6 significantly decreased, while the others either did not change such as TNFα and IFNɣ, or even increased such as IL1β (**Figure 2E**). These results suggest that microglia in AIS may undergo an incomplete phenotypic switch to M2 polarization. Subsequently, we compared the expression levels of Netrin-1 and its primary receptors, DCC, UNC5a, UNC5b, UNC5d and A2B, in microglia from the two groups. We detected a significant upregulation of Netrin-1 and UNC5a, but not other Netrin-1 receptors, in microglia at day 3 after ischemia induction compared to those from sham treatment (**Figure 2E**), consistent with our observations in clinical specimens. Therefore, our results suggest that cerebral microglia from experimental rodents with ischemic stroke exhibit an increased expression of Netrin-1 and its receptor UNC5a.

### Generation of plasmids expressing Netrin-1 specifically in microglia

**Figure 3:**
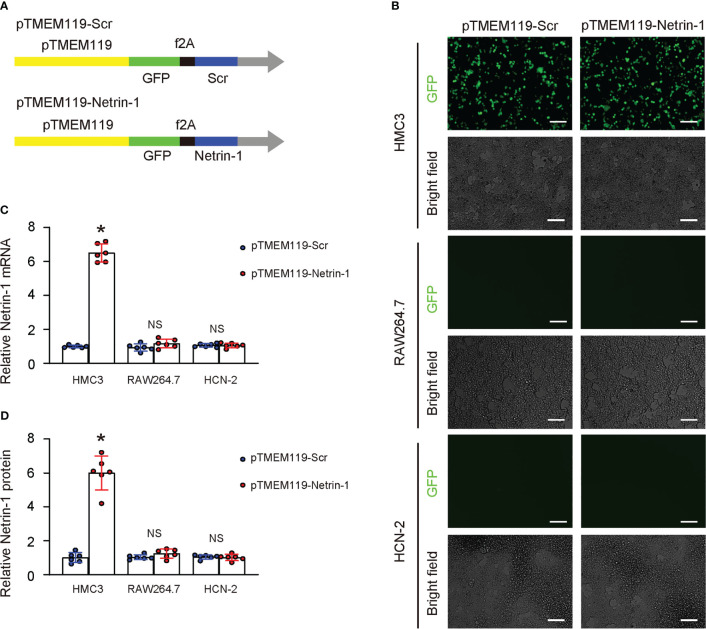
Generation of plasmids expressing Netrin-1 specifically in microglia. **(A)** Schematic of a designed plasmid that utilized a microglia-specific TMEM119 promoter to drive the Netrin-1 transgene or a scramble sequence (Scr). Both plasmids included a green fluorescent protein (GFP), also controlled by the TMEM119 promoter and linked to the transgene by an f2A sequence. **(B–D)** The plasmids were transfected into a mouse microglia cell line (HMC3), a mouse macrophage cell line (RAW264.7), and a mouse neuronal cell line (HCN-2). **(B)** GFP and bright field channels for the transfected cells in culture. **(C, D)** RT-qPCR **(C)** and ELISA for Netrin-1 in transfected cells. *p<0.05. NS, no significance. Scale bars were 50µm.

To investigate the effects of Netrin-1 on microglia, we designed a plasmid that utilized a microglia-specific TMEM119 promoter to drive the Netrin-1 transgene or a scramble sequence (Scr) (13). Both plasmids included a green fluorescent protein (GFP), also controlled by the TMEM119 promoter and linked to the transgene by an f2A sequence (**Figure 3A**). The TMEM119 promoter was chosen due to the potential application of these constructs for the specific targeting of microglia *in vivo*. The plasmids were transfected into a mouse microglia cell line (HMC3), a mouse macrophage cell line (RAW264.7), and a mouse neuronal cell line (HCN-2). The specificity of the TMEM119 promoter for microglia was confirmed by the observation that only HMC3 cells were green fluorescent after transfection (**Figure 3B**). Additionally, the transfection of RAW264.7 or HCN-2 cells with either plasmid did not result in a significant alteration of Netrin-1 mRNA (**Figure 3C**) or protein (**Figure 3D**) levels, while the transfection of HMC3 with pTMEM119-Netrin-1 significantly increased Netrin-1 mRNA (**Figure 3C**) and protein (**Figure 3D**) levels, thus validating the efficacy of the microglia-specific Netrin-1-modifying plasmids.

### Netrin-1 increases M2-like polarization of microglia *in vitro* through UNC5a

**Figure 4:**
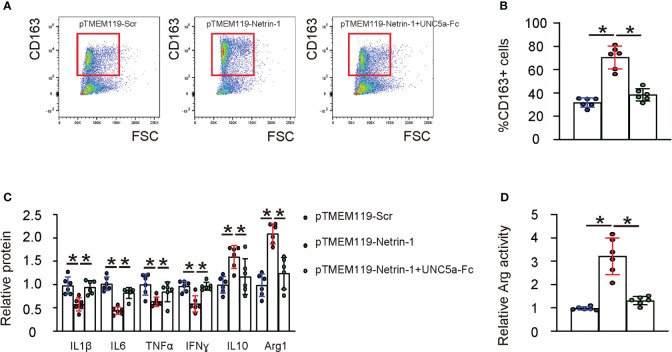
Netrin-1 increases M2-like polarization of microglia *in vitro* through UNC5a. HMC3 cells were transfected with pTMEM119-Netrin-1 and pTMEM119-Scr plasmids *in vitro*. An experimental condition was included for which UNC5a-Fc was also added to block the Netrin-1 signaling through the UNC5a receptor in the transfected cells. **(A)** Representative flow charts for CD163 in the transfected cells. **(B)** Quantification of CD163-postive cell percentage. **(C)** ELISA for IL-1β, IL-6, IFNɣ, TNFα, IL-10 and arginase 1 (ARG1) in the transfected cells. **(D)** Arginase assay. *p<0.05.

To examine the effect of Netrin-1 on the M1/M2 polarization of microglia, we transfected HMC3 cells with pTMEM119-Netrin-1 and pTMEM119-Scr plasmids *in vitro*. Based on our clinic data and bioinformatic analysis of a public database, which suggested that microglial Netrin-1 may influence ischemic stroke through UNC5a, we also included an experimental condition in which UNC5a-Fc was added to block the Netrin-1 signaling through the UNC5a receptor in the transfected cells. Flow cytometry was performed to examine the M2 marker CD163 in the transfected cells, revealing that pTMEM119-Netrin-1 transfection significantly increased the CD163-positive cells (**Figures 4A, B**). Furthermore, transfection with pTMEM119-Netrin-1 significantly reduced the proinflammatory factors IL-1β, IL-6, IFNɣ and TNFα, and significantly increasing the levels of anti-inflammatory factors IL-10 and arginase 1 (ARG1) (**Figure 4C**) and the arginase activity of microglia (**Figure 4D**). Notably, all these effects were significantly attenuated in the presence of UNC5a-Fc (**Figures 4A–D**), indicating that Netrin-1 reduces the M2-like polarization of microglia *in vitro* through UNC5a.

### Netrin-1 reduces apoptosis and invasiveness of microglia *in vitro* through UNC5a

**Figure 5:**
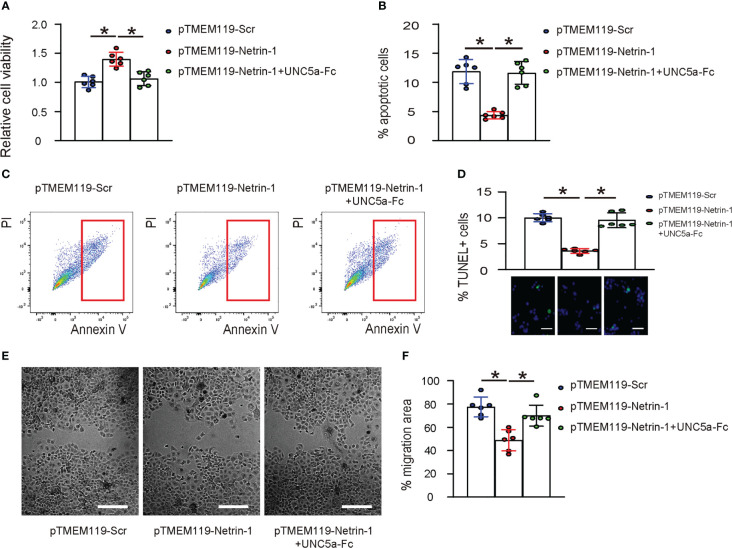
Netrin-1 reduces apoptosis and invasiveness of microglia *in vitro* through UNC5a. **(A)** CCK-8 assay to assess the viable cells of pTMEM119-Netrin-1- and pTMEM119-Scr- transfected microglia in hypoxia condition, with/without presence of UNC5a-Fc. **(B)** Analysis of apoptotic cell death by Annexin V assay, shown by quantification **(B)** and by representative flow charts **(C)**. **(D)** TUNEL staining shown by quantification and representative images. **(E, F)** Scratch cell migration assay, shown by representative images **(E)** and by quantification **(F)**. *p<0.05. Scale bars were 50µm.

The survival of pTMEM119-Netrin-1- and pTMEM119-Scr- transfected microglia was examined in hypoxia condition. We found that transfection with pTMEM119-Netrin-1 significantly increased their viability (**Figure 5A**), likely through reduction in their apoptotic cell death (**Figures 5B–D**), and significantly reduced their invasion and migration (**Figures 5E, F**). Notably, all these effects were significantly attenuated in the presence of UNC5a-Fc (**Figures 5A–F**), indicating that Netrin-1 reduces apoptosis and invasiveness of microglia *in vitro* through UNC5a.

### AAVPHP.B-pTMEM119-Netrin-1 alleviates experimental ischemic stroke in mice

**Figure 6:**
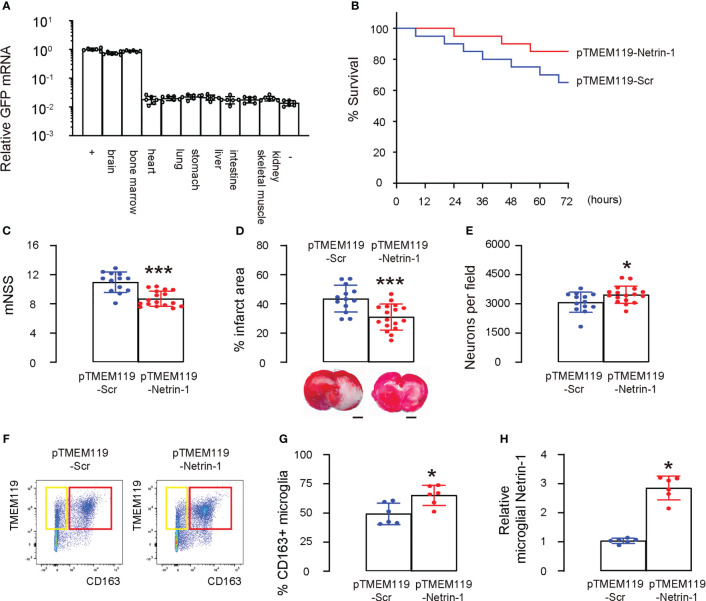
AAVPHP.B-pTMEM119-Netrin-1 alleviates experimental ischemic stroke in mice. Two AAV vectors AAVPHP.B-pTMEM119-Netrin-1 and AAVPHP.B-pTMEM119-Scr were created to allow specifically express Netrin-1 or Scr in microglia *in vivo* during a gene therapy approach. **(A)** To ensure that the AAVs could cross the blood-brain barrier, we chose a specific serotype, PHP.B (14–16), and administered the AAVs *via* the tail vein of mice. RT-qPCR for GFP in mouse tissue, including brain, bone marrow, heart, lungs, stomach, liver, intestines, skeletal muscle and kidneys. “+” is the HMC3 transfected with GFP plasmids as a positive control, while “-” is negative control of innocent mouse brain tissue. **(B)** The 72 hours’ survival curve of mice after MCAO. Each group had 20 mice. **(C)** At 72 hours after reperfusion, mouse neurological function was assessed by mNSS. **(D)** Quantification of the infarct volume and representative images in the brain. **(E)** The neuronal content in mouse brain. **(F)** Microglia were isolated from the mouse brain by flow cytometry, shown by representative flow charts **(F)** and by quantification **(G)**. **(H)** ELISA for microglial Netrin-1. *p<0.05. ***p<0.001. Scale bars were 2mm.

We created two AAV vectors AAVPHP.B-pTMEM119-Netrin-1 and AAVPHP.B-pTMEM119-Scr that specifically express Netrin-1 or Scr in microglia *in vivo* using our microglia-targeting plasmids. To ensure that the AAVs could cross the blood-brain barrier, we chose a specific serotype, PHP.B (14–16), and administered the AAVs *via* the tail vein of mice. GFP mRNAs were detected exclusively in the mouse brain and bone marrow, but not in other organs, including the heart, lungs, stomach, liver, intestines, skeletal muscle, and kidneys, confirming both the ability of the AAV serotype PHP.B to cross the blood-brain barrier and the specificity of the TMEM119 promoter for microglia (**Figure 6A**). Afterwards, we investigated whether administering AAVPHP.B-pTMEM119-Netrin-1 to mice could improve their survival and protect against neuron loss following ischemic stroke. Mice were given AAVPHP.B-pTMEM119-Netrin-1 or AAVPHP.B-pTMEM119-Scr three days before inducing ischemic stroke by MCAO, and after reperfusion for 72 hours, the mice were analyzed. Firstly, we found that AAVPHP.B-pTMEM119-Netrin-1 significantly increased the survival of mice after MCAO (**Figure 6B**). At 72 hours after reperfusion, AAVPHP.B-pTMEM119-Netrin-1-treated mice exhibited significantly better neurological function as assessed by mNSS (**Figure 6C**) and significantly smaller infarct volume in the brain (**Figure 6D**). The neuronal content in mouse brain was quantified at sacrifice, showing a significant reduction in the loss of neurons in AAVPHP.B-pTMEM119-Netrin-1-treated mice compared to AAVPHP.B-pTMEM119-Scr mice (**Figure 6E**). Microglia were isolated from the mouse brain, showing significant increases in M2 polarization of microglia (**Figures 6F, G**) and the levels of microglial Netrin-1 (**Figure 6H**). Taken together, these data suggest that AAVPHP.B-pTMEM119-Netrin-1 can alleviate experimental ischemic stroke in mice, likely by modulating microglial phenotype.

## Discussion

Netrin-1 is known to signal through its receptors UNC5H (UNC5a, UNC5b, UNC5d) and DCC, but their specific signaling mechanisms are unclear. DCC has been suggested to function as a dependence receptor, which induces apoptosis when unoccupied by ligand (17). However, this potential of inducing apoptosis is abolished in the presence of ligand (17). Conversely, all UNC5a, UNC5b, UNC5d display a death domain, and their expression induces apoptosis while the presence of netrin-1 blocks this effect, indicating that they may also function as dependence receptors (18). UNC5H proteins are also caspase substrates, and its caspase-mediated cleavage causes apoptotic cell death (18). Netrin-1 and its receptor signaling play a critical role during nervous system development (19). In our study, the role of microglial Netrin-1 in ischemic stroke was investigated, and we found that Netrin-1 improved the survival of microglia in hypoxia, possibly due to its binding to UNC5H, with UNC5a being the most important, which subsequently blocked the function of the freely released death domains of UNC5H.

Our study revealed a similar M2-like phenotypic adaptation in human and mouse microglia during ischemic stroke. However, the data from a rat study in a public database suggested that the polarization may be incomplete (20), which could be attributed to species differences and the limited number of repeats in the rat microglial array data (20). To overcome off-target effects of most current drug therapies, we employed a microglia-specific gene targeting approach and a delivery system that allowed crossing the blood-brain barrier in a mouse model (21). This method has been validated in *in vitro* and *in vivo* studies and holds great promise for clinical applications (21).

Here, activation of Netrin-1 receptor signaling in microglia, mainly through its receptor UNC5a, has been found to result in various effects on microglia that ultimately exert protective effects on neuronal cells *in vivo*. This protective effect may be due to several factors. Firstly, the M2-like polarized microglia may reduce the production and release of proinflammatory cytokines, thereby reducing damage to neurons (22). Secondly, the reduced migratory potential of microglia may contribute to the resolution of immune responses in the microenvironment (23). Lastly, the improved survival of microglia could attenuate the infiltration of inflammatory cells from circulation, ultimately promoting the termination of inflammation.

Previous studies have suggested that M2-like macrophages increase their motility and migration (24), which was not observed in Netrin-1-expressing microglia. This may be due to the comparison of M2-polarized microglia expressing Netrin-1 to M1-like microglia rather than M0 quiescent microglia (25), since M1 microglia are known to have high motility and migratory potential due to the expression of CCR2 and CXCR3 (26–28). Additionally, macrophages/microglia are a heterogeneous group of cells that can exhibit different phenotypes based on their environment, and measuring their exact motility and migration can be challenging due to a lack of standardized degree of their polarization (29).

Together, our study suggests that Netrin-1 and its receptors could be potential therapeutic targets for promoting post-ischemic survival and functional recovery. Further research is needed to elucidate the specific signaling mechanisms of Netrin-1 through its receptors and to explore the potential of targeting Netrin-1 in the treatment of ischemic stroke.

## Data availability statement

The original contributions presented in the study are included in the article/Supplementary Material. Further inquiries can be directed to the corresponding authors.

## Ethics statement

The studies involving human participants were reviewed and approved by Shanghai Ninth People’s Hospital. The patients/participants provided their written informed consent to participate in this study. The animal study was reviewed and approved by Shanghai Ninth People’s Hospital.

## Author contributions

XY, YaL, WZhong, YiL and WZhang were responsible for data collection, analysis and interpretation. XY designed the projected, obtained the funding and wrote the manuscript. All authors proved the manuscript for publication.
